# Arrhythmogenic Right Ventricle in Left Ventricular Non-compaction - In Response to the Letter to the Editor "Diagnostic Dilemmas for Underlying Pathophysiology of Arrhythmias Originating from the Right Ventricle"

**DOI:** 10.1016/s0972-6292(16)30739-2

**Published:** 2014-03-12

**Authors:** Shohreh Honarbakhsh, Irina Suman-Horduna, Lilian Mantziari, Sabine Ernst

**Affiliations:** 1 Royal Brompton and Harefield NHS Foundation Trust, United Kingdom; 2 NIHR Cardiovascular Biomedical Research Unit, Royal Brompton and National Heart and Lung Institute, Imperial College London

**Keywords:** Right Ventricular Tachycardia, Left Ventricular Non-compaction Cardiomyopathy

 We would like to thank the author for their interest in our manuscript [1] as well as for their considerate comments. Our patient initially presented in another center with ventricular ectopics (VEs) that were mapped and ablated from the right ventricular outflow tract. The second arrhythmia foci had left bundle branch block morphology but different axis, and it was mapped and ablated by us at the base of the RV towards the infero-lateral aspect of the tricuspid annulus. We share the authors' opinion that in patients with multiple arrhythmia foci from different parts of the RV myocardium ARVD/C should be considered and notably, that was our initial presumptive diagnosis; however, other features should be identified to further support the diagnosis or ARVD/C. The existing diagnostic criteria are based on major and minor points including structural parameters identified on cardiac magnetic resonance (CMR) or 2D echocardiogram, histological features on myocardial biopsy, depolarization or repolarization abnormalities on ECG, often occurring in a context of familial disease [2].

 In our case we successfully ablated an arrhythmia focus originating from a normally appearing RV free wall in a patient in whom the transthoracic echocardiogram and cardiac magnetic resonance (CMR) showed features consistent with left ventricular non-compaction cardiomyopathy (LVNC). On the steady state precession cines and late gadolinium enhancement images on the CMR there was no features of RV dilatation, regional wall motion abnormality or reduced systolic function (ejection fraction or fractional shortening) which are all diagnostic criteria seen in ARVD/C. Moreover, the CMR did not show any features of intra-myocardial fatty infiltration or replacement fibrosis, RV hypertrophy or localized aneurysm formation that is further reported in this cohort of patients [3].

 Likewise the 12 lead ECG in our patient showed no repolarization abnormalities that can be seen in ARVD/C. We appreciate that a non-specific or normal appearing ECG does not always preclude ARVD/C diagnosis, however in the study by Te Riele et al. [4] quoted by the authors the patients with non-specific or normal ECG had alternative evidence of disease expression, which was not the case in our patient. Even though there are recessive forms of ARVC/D, more frequently, this condition has autosomal dominant inheritance. Our patient had no family history of either diagnosed or possible undiagnosed ARVD/C, further making ARVDC/C unlikely in our patient.

 Despite the fact that the RV was macroscopically normal, in our LVNC case the RV did have an arrhythmogenic potential. Because of the difficulty in distinguishing normal variants in the highly trabeculated RV from the pathological non-compacted ventricle, the existence of RV non-compaction is disputed by some authors [5,6]. Whether the RV arrhythmogeneity is part of the clinical picture of a patient with LVNC, or two independent conditions - LVNC and ARVD/C - occurred simultaneously in the same patient such as reported by Song ZZ et al [7], remains an unresolved issue. Although we entirely agree with the authors' statement that an alternative diagnosis of ARVD/C should be sought in similar situations, we believe that in our case the burden of evidence did not support a co-diagnosis of ARVD/C.
